# Whole-Cell Display of Phosphotransferase in *Escherichia coli* for High-Efficiency Extracellular ATP Production

**DOI:** 10.3390/biom12010139

**Published:** 2022-01-15

**Authors:** Shuai Zhao, Guoli Yang, Xiaochen Xie, Guangbo Yan, Fei Wang, Wanping Chen, Lixin Ma

**Affiliations:** State Key Laboratory of Biocatalysis and Enzyme Engineering, Hubei Key Laboratory of Industrial Biotechnology, School of Life Sciences, Hubei University, Wuhan 430062, China; zhaoshuai199625@163.com (S.Z.); yangguoli9494@163.com (G.Y.); xiexc@stu.hubu.edu.cn (X.X.); 17607164502@163.com (G.Y.)

**Keywords:** ATP regeneration, visible sfGFP, PAP-Pb, cell-surface display, reused

## Abstract

Adenosine triphosphate (ATP), as a universal energy currency, takes a central role in many biochemical reactions with potential for the synthesis of numerous high-value products. However, the high cost of ATP limits industrial ATP-dependent enzyme-catalyzed reactions. Here, we investigated the effect of cell-surface display of phosphotransferase on ATP regeneration in recombinant *Escherichia coli*. By N-terminal fusion of the super-folder green fluorescent protein (sfGFP), we successfully displayed the phosphotransferase of Pseudomonas brassicacearum (PAP-Pb) on the surface of *E. coli* cells. The catalytic activity of sfGFP-PAP-Pb intact cells was 2.12 and 1.47 times higher than that of PAP-Pb intact cells, when the substrate was AMP and ADP, respectively. The conversion of ATP from AMP or ADP were up to 97.5% and 80.1% respectively when catalyzed by the surface-displayed enzyme at 37 °C for only 20 min. The whole-cell catalyst was very stable, and the enzyme activity of the whole cell was maintained above 40% after 40 rounds of recovery. Under this condition, 49.01 mg/mL (96.66 mM) ATP was accumulated for multi-rounds reaction. This ATP regeneration system has the characteristics of low cost, long lifetime, flexible compatibility, and great robustness.

## 1. Introduction

Adenosine-5’-triphosphate (ATP), is one of the essential molecules in living systems [1], plays a central role in many biochemical reactions, and has the potential to synthesize many high-value products [2,3]. The synthesis and consumption of ATP play an important role in many aspects of cell metabolism, such as active transport mechanisms [1,2], ATP-binding cassette (ABC) transporters [4], and ATP as the precursor to synthesize DNA, RNA, and NAD(P) [5]. ATP is necessary for the biosynthesis of cyclic adenosine monophosphate (cAMP), that is a significant second messenger in signal transduction; ATP also can serve as a signal ligand for ATP-sensitive or purinergic ionotropic and G-protein coupled receptors [6]. ATP provides energy for the biosynthesis of a large number of biological compounds, such as amino acids, proteins, and lipids [6]. Large amounts of ATP are also needed in another important field that is cell-free protein expression, as they are involved in complex reaction cascades [1,7,8], such as the production of S-adenosyl-homocysteine (SAH), glutathione (GSH), and S-adenosyl-methionine (SAM) [9].

Traditional ATP synthesis is chemically synthesized from the substrate AMP or ADP with compounds with phosphoric acid groups as the phosphorylation reagents [10]. In some cases, ATP was regenerated by the direct transfer of a phosphoryl group of another phosphorylated compound to ADP and AMP for energy metabolite [2]; this is the most significant way of ATP regeneration for anaerobic microorganisms and cells during anoxia [2]. It provides a quicker, less energy-efficient source of ATP, and it does not require the FoF1 ATP synthase driven by proton motive forces (PMF) across the membranes compared with oxidative phosphorylation and photophosphorylation to regenerate ATP [5,11]. At the same time, there are other types of ATP regeneration, such as co-production of glutathione and S-adenosyl-methionine [9].

The enzyme AMP phosphotransferase of Pseudomonas brassicacearum (PAP-Pb) can catalyze the phosphorylation of nucleotides by using polyphosphates [poly(P)] as phosphate donors [12]. It catalyzes the conversion of the terminal phosphate residue of poly(P) to AMP, resulting in the synthesis of ADP [3,12]. Recently, research has found that PAP-Pb can also use the ADP as a substrate to form ATP [13].

Proteins or peptides can be displayed on the surface of bacterial cells. This method is widely used in research and industry, such as antigen or antibody epitope analysis [14]. As we known, protein secretion is always conferred by a signal peptide that can target the protein to specific secretory pathways [15,16], such as the N-terminus of ice nucleation protein (INP) [17,18] and N-terminal domain of intimin fusions [19]. Because *E. coli* is a Gram-negative bacterium, which has a cell envelope with two biological membranes such as the cytoplasmic inner membrane and the outer membrane [11], the presence of the outer membrane is the main obstacle to display related proteins on the surface of *E. coli*.

Cell-surface display allows peptides and proteins to be displayed on the surface of microbial cells by fusing them with the anchoring motifs [20]. To display an enzyme on the surface of a living cell bears several advantages. First, both the substrate and the product of the enzymatic reaction do not need to cross a membrane barrier [21], so the whole cell biocatalyst exhibits high efficient biodegradation than the purified enzyme [22,23]. Second, the enzyme being linked to the cell can be separated from the reaction mixture by simple centrifugation, and thus separated from the product it can be transferred to a new substrate preparation resulting in multi-rounds of enzymatic conversion [21], enhancing the catalytic efficiency and stereoselectivity [24,25], reducing reaction costs. Finally, the anchoring in a matrix, in this case, the cell envelope stabilizes the enzyme and makes it less accessible to proteolytic degradation and material adsorption resulting in continuous higher activities [21], so the whole cell biocatalyst exhibited better stability than the purified enzyme [22,26]. Whole-cell biocatalysts that display enzymes on the cell surface have become a solution to specific biotechnological problems and already applied in industrialization.

Super-folder green fluorescent protein (sfGFP) has a stable beta-barrel structure and emits green fluorescent light when it is exposed to the light ranging from blue to ultraviolet spectral spectrum [27]. The high solubility, obvious green fluorescent, and fast folding ability of the sfGFP protein [15], make the sfGFP protein as a guide to secret target protein to the outer membrane [19,27]. It has been recently reported that sfGFP can serve as a non-signal peptide to guide protein automatic secretion in *E. coli* due to its beta-barrel structure, net negative charges, and fast folding ability [19,27].

Super-folder green fluorescent protein (sfGFP) comes with fluorescence and can be viewed directly without equipment [27,28]. So, when expressing the target protein fusion with sfGFP tag, we can directly, from the fluorescence, judge the presence of secreted sfGFP-fusion protein in cells supernatant or in the medium. The sfGFP expression system can not only achieve the rapid screening of high-level expression strains by detecting fluorescence, but also achieve the secretion of target proteins [29,30]. It has been reported that sfGFP as fusion tag not only facilitates the solubility but also promotes the stability of target protein in *Escherichia coli* [28].

**Figure 1:**
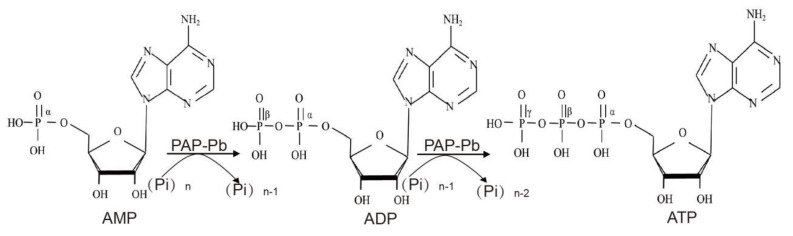
Schematic presentation of polyphosphate-based ATP regeneration from AMP or ADP catalyzed by phosphotransferase.

Here, we present a successful cell-surface display system of PAP-Pb in recombinant *E. coli* by N-terminal fused with the sfGFP protein and used the intact cells to effectively synthesize ATP. In this process, we performed the ATP regeneration system for ATP biosynthesis with only one enzyme, AMP or ADP can be phosphorylated into ATP (Figure 1). The sfGFP-PAP-Pb displayed intact cells as whole-cell catalysts are convenient to achieve ideal conversion yield without protein extraction. To investigate the effect of the display of PAP-Pb on ATP production, we tested the PAP-Pb-displaying recombinant *E. coli* in various environmental conditions.

## 2. Materials and Methods

### 2.1. Strains, Plasmids, and Chemical Reagents

*Escherichia coli* BL21(DE3) and DH5α were stored in the laboratory. The pET-23a(+), pET-23a(+)-sfGFP [31] used as the expression vector were maintained in our laboratory. The plasmid pET-28a(+)-PAP-Pb coding phosphotransferase for Pseudomonas brassicacearum (PAP-Pb) was synthesized by Wuhan GeneCreate Biological Engineering Co., Ltd. (Wuhan, China). Competent *E. coli* DH5α and *E. coli* BL21(DE3) were prepared in the laboratory. Adenosine triphosphate (ATP), adenosine diphosphate (ADP), and adenosine monophosphate (AMP) were purchased from the Shanghai Macklin, and polyphosphates [poly(P)] was purchased from Macklin. The primers used in the experiments (Table 1) were synthesized by Shanghai Sangon Biological Engineering Technology & Services Co., Ltd. (Shanghai, China).

### 2.2. Construction of Recombinant Plasmids for Cell Surface Display

**Figure 2:**
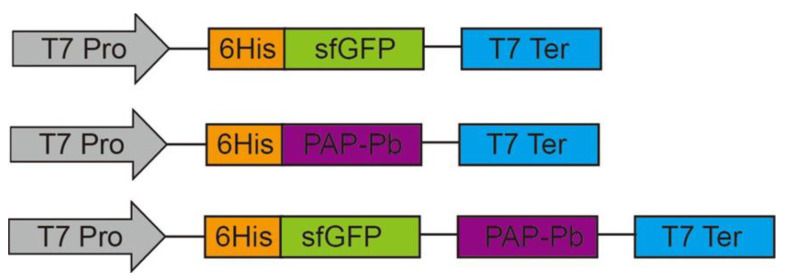
Construction of recombinant plasmids for the cell surface display of PAP-Pb. T7 Pro: T7 promoter; T7 Ter: T7 terminator.

The PAP-Pb (WP_013692547.1.) was synthesized and cloned into the vector pET-23a(+) and pET-23a(+)-sfGFP as shown in Figure 2. The recombinants were identified by sequencing.

### 2.3. Secretory Expression of the Target Protein

The plasmids and strains used in this research are shown in Table 2. The recombinants pET23a-PAP-Pb and pET23a-sfGFP-PAP-Pb were transformed into *E. coli* BL21(DE3) competent cells. One of the positive clones was inoculated into 5 mL LB medium with 50 μg/mL Ampicillin and cultured at 220 rpm and 37 °C. Then the overnight cultures were transferred to 100 mL LB medium with 50 μg/mL ampicillin and cultured at 220 rpm and 37 °C. When the optical density at 600 nm (OD_600_) reached to 0.7–1.6, cells were harvested by centrifugation (15,000× *g*, 5 min, 4 °C ), the pellet was completely resuspended in 10 mL of phosphate-buffered saline (PBS, pH 7.4) and then treated with ultrasonication. After centrifugation at 15,000× *g* for 10 min, the supernatant and pellet were analyzed with SDS-PAGE.

### 2.4. Cell Fractionation

Cell fractionation was performed according to the method described by Quan [32,33]. Cells were harvested from 10 mL culture broth (OD600 ≈ 1.6) by centrifugation (5000 g, 10 min, 4 °C) and treated with one-third volume of Tris-EDTA-NaCl solution (TEN) containing 50 mM Tris-HCl, 5 mM EDTA, and 50 mM NaCl (pH 8.0). The mixture was incubated at 4 °C overnight processing [11], then the solution was centrifuged at 5000 g at 4 °C for 20 min. The supernatant was regarded as outer membrane fraction, and then supernatant was collected, and protein samples were detected by 10% SDS-PAGE.

### 2.5. Fluorescence Microscopy

Cells were harvested from 1 mL culture broth (OD_600_ ≈ 1.6) by centrifugation (3000× *g*, 5 min, 4 °C), and resuspended with 20 mL phosphate buffer. Then 5 µL cells were pipetted onto Poly-prep microscopy slides and studied in a Zeiss LSM 980 fluorescence, using ZEN v3.0 for capturing images.

### 2.6. Biosynthesis of ATP

The 1 mL reaction mixture used for the synthesis of ATP containing 20 mM poly(P), 4 mM ADP or AMP, 30 mM Mg^2+^, 50 mM Tris-HCl (pH 8.5), and appropriate amounts of PAP-Pb or sfGFP-PAP-Pb (supernatant or intact cells). The reaction was performed in a shaker at 37 °C and terminated by heating the mixture at 80 °C for 10 min, followed by detecting the products with high-performance liquid chromatography (HPLC, Welch) after diluting five-fold with sterile dH_2_O.

### 2.7. Detection and Analysis by High-Performance Liquid Chromatography (HPLC)

ATP, ADP, and AMP were monitored with HPLC (Welch), equipped with a Welch Ultimate LP-C18 column (4.6 × 250 mm, 5 μm, Welch Materials, Inc, Shanghai, China) after 0.22 μm filtration. The mobile phase was methanol with 50 mM ammonium formate buffer (pH 4.5) (95% *v/v*) at a flow rate of 0.5 mL/min [34], and the effluent was detected at a wavelength of 260 nm. The detection times were as follows: ATP (7.453 s), ADP (8.489 s), and AMP (14.009 s).

### 2.8. Effect of pH and Mg^2+^ Concentration ATP Synthesis

A single variable was maintained to determine the effect of the optimum pH and Mg^2+^ concentration. The reaction mixture was prepared as previously described. The optimal pH for the reaction was determined by experiments using a pH range of 7.0–9.0 at 37 °C with the buffer of 20 mM poly(P), 4 mM ADP or AMP, 30 mM Mg^2+^, 50 mM Tris-HCl; the optimal Mg^2+^ concentration was determined using the concentration from 0–100 mM at 37 °C with the buffer of 20 mM poly(P), 4 mM ADP or AMP, 50 mM Tris-HCl (pH 8.5). All experiments were performed for at least three biological replicates.

### 2.9. Effect of Polyphosphate Concentration on ATP Synthesis

A single variable was maintained to determine the effect of the optimum polyphosphate concentration. The reaction mixture was prepared as previously described. The optimal polyphosphate concentration was determined using the concentration from 0–150 mM at 37 °C with the buffer of 4 mM ADP or AMP, 30 mM Mg^2+^, 50 mM Tris-HCl (pH 8.5). The experiment was performed for at least three biological replicates.

### 2.10. Effect of the Cell State on the Production of ATP

The recombinant pET23a-sfGFP-PAP-Pb was transformed into *E. coli* BL21(DE3) competent cells. One of the positive clones was inoculated into 5 mL LB medium with 50 μg/mL ampicillin and cultured at 220 rpm and 37 °C. Then the overnight cultures were transferred to 100 mL LB medium with 50 μg/mL ampicillin and cultured at 220 rpm and 37 °C until the OD_600_ reached to 0.7. The OD_600_ was measured every hour until OD_600_ reached to 1.7, then the OD_600_ was measured every 5 h. The same amount of cells with different OD_600_ were used in the reaction system using AMP or ADP as substrates to detect the enzyme activity, respectively.

### 2.11. Effects of Temperature and Stability of Whole-Cell Catalysts

The optimal temperature was determined using the temperature range of 18–50 °C at optimal pH and Mg^2+^ concentration. The reaction mixture was prepared as previously described, containing 20 mM poly(P), 4 mM ADP or AMP, 30 mM Mg^2+^, 50 mM Tris-HCl (pH 8.5) in 1 mL reaction mixture.

For detecting the stability of whole-cell catalysts, the same amount of cells were incubated at 37 °C, 40 °C, and 45 °C for different lengths of time. Then they were used in the reaction system using AMP or ADP as substrates to detect the residual activity of the enzyme.

### 2.12. Reuse of Whole-Cell Catalyst

The whole-cell catalyst was tested at optimal pH, temperature, Mg^2+^, and poly(P) concentration. The 1 mL reaction mixture contains 20 mM poly(P), 4 mM ADP or AMP, 30 mM Mg^2+^, 50 mM Tris-HCl (pH 8.5), and appropriate amounts of sfGFP-PAP-Pb intact cells. After reaction at 37 °C for 20 min, the reaction mixture was centrifuged at 15,000 g for 5 min, and the pellet was used for the next reaction.

## 3. Results

### 3.1. Expression of the Recombinant Proteins

**Figure 3:**
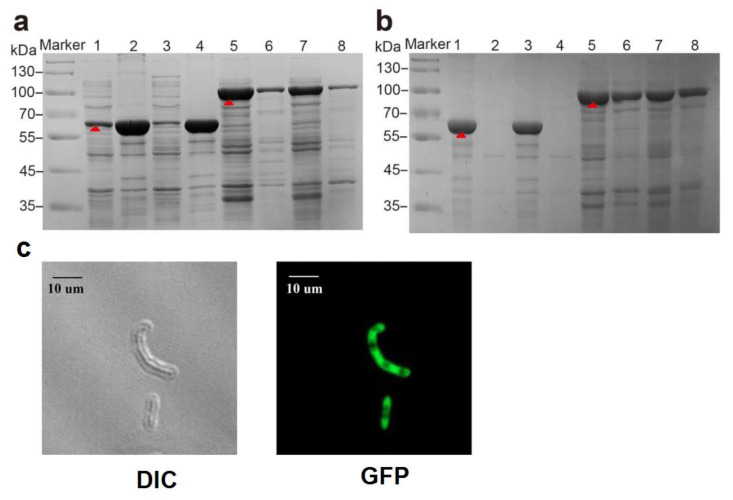
Analysis of PAP-Pb and sfGFP-PAP-Pb. (**a**): SDS-PAGE analysis of the protein expression. Lane 1: supernatant of the cell lysate of PAP-Pb without induction; lane 2: precipitate of PAP-Pb cell lysate without induction; lane 3: supernatant of PAP-Pb cell lysate after induction for 10 h; lane 4: precipitate of PAP-Pb cell lysate after induction 10h; Lane 5: supernatant of the cell lysate of sfGFP-PAP-Pb without induction; lane 6: precipitate of sfGFP-PAP-Pb cell lysate without induction; lane 7: supernatant of sfGFP-PAP-Pb cell lysate after induction 10 h; lane 8: precipitate of sfGFP-PAP-Pb cell lysate after induction for 10 h. The red arrow indicates the PAP-Pb or sfGFP-PAP-Pb. (**b**): SDS-PAGE analysis of outer membrane cell fraction: Lanes 1–2: the total cell and the outer membrane fraction of PAP-Pb without induction; lanes 3–4: the total cell and the outer membrane fraction of PAP-Pb after induction; lanes 5–6: the total cell and the outer membrane fraction of sfGFP-PAP-Pb without induction; lanes 7–8: the total cell and the outer membrane fraction of sfGFP-PAP-Pb after induction. The red arrow indicates the PAP-Pb or sfGFP-PAP-Pb. (**c**): Confocal laser microscopy was used to detect the distribution of sfGFP-PAP-Pb protein in *E. coli* BL21(DE3). DIC and GFP represent the images obtained by scanning in bright and dark fields, respectively.

Recombinant strains E-PAP-Pb and E-sfGFP-PAP-Pb were cultured as described in the methods section. Proteins were extracted and analyzed by SDS-PAGE. As shown in Figure 3a, the PAP-Pb (61.0 kDa)was mainly detected in the precipitate of the cell lysate whether it was induced, while the fusion protein sfGFP-PAP (87.0 kDa)was mainly observed in the supernatant of the cell lysate. As indicated, the sfGFP-PAP-Pb was more soluble compared to PAP-Pb. The results indicate sfGFP enables proper folding of PAP-Pb proteins in *E. coli*.

To further verify whether PAP-Pb and sfGFP-PAP-Pb were successfully displayed on the cell surface, the cell fraction of outer membrane was separated and analyzed via SDS-PAGE. As shown in Figure 3b, sfGFP-PAP-Pb mainly appeared in the fraction of the outer cell membrane. While few of PAP-Pb was detected in the fraction of the outer cell membrane. This is consistent with previous reports that sfGFP can promote protein secretion outside the cell membrane [27,28].

Laser scanning confocal microscopy (LSCM) was used to detect the distribution of sfGFP-PAP-Pb in *E. coli* BL21(DE3) without any dyeing process. Under the eyepiece, the objective lens magnification was adjusted to find the cells that needed to be observed. The LSCM was switched to scan mode, and the laser intensity parameters were adjusted to obtain a clear confocal image. The images obtained by scanning in the bright-field and dark-field fields of view are shown in Figure 3c. It was observed that sfGFP-PAP-Pb bacterial cells had bright autofluorescence, and sfGFP could also be found in the background.

### 3.2. Biosynthesis of ATP with Fusion Enzyme Displayed on the Surface of E. coli

**Figure 4:**
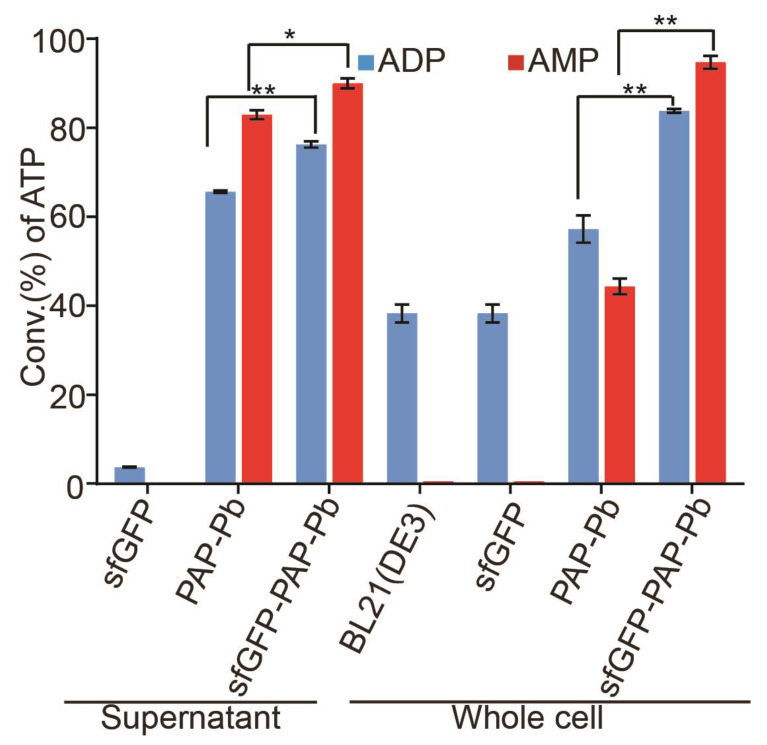
Analysis of ATP conversation with crude enzyme and intact cells. The reaction mixture contains 20 mM poly(P), 4 mM ADP or AMP, 30 mM Mg^2+^, and 50 mM Tris−HCl buffer (pH 8.5). The total volume was brought up to 1000 μL with double-distilled water. Different enzymes or cells were added as shown, sfGFP and BL21(DE3) were as negative controls. The reaction solution was incubated at 37 °C for 20 min. A small amount of reaction solution was terminated in 80 °C water bath for 10 min and diluted five-fold for HPLC analysis. Results are means ± SD of three parallel replicates (* *p* < 0.05, ** *p* < 0.01).

We cultured *E. coli* BL21(DE3) strains harboring psfGFP-PAP-Pb and pPAP-Pb plasmid to generate ATP, respectively. The strain containing psfGFP was used as a control. To ensure the same number of cells were applied in the reaction, the cells expressed with sfGFP-PAP-Pb, PAP-Pb or sfGFP were collected and diluted to the same OD_600_. To evaluate the effect of the cell-surface display of PAP-Pb, we compared the conversion rate of ATP with displayed sfGFP-PAP-Pb and cytosol PAP-Pb. As shown, the fusion sfGFP-PAP-Pb possessed better catalytic activity than the PAP-Pb (Figure 4). For the supernatant of sfGFP-PAP-Pb and PAP-Pb, the conversion rate of ATP catalyzed by sfGFP-PAP-Pb was higher than that by PAP-Pb whether the substrate was AMP (*p* < 0.05) or ADP (*p* < 0.01), that is consistent with the protein expression level shown in Figure 3a. However, for the intact cells, the conversion rate of ATP catalyzed by sfGFP-PAP-Pb was significantly higher than that of PAP-Pb whether the substrate was AMP or ADP (*p* < 0.01). The results demonstrate the advantage of the cell-surface display to improve productivity [35]. The increased productivity may be due to a shortened ATP reaction step. In the cytosol-expressed PAP-Pb strain, AMP or ATP needs to be transported into the cell to react with PAP-Pb and be converted to ATP. Then, ATP is secreted back to the extracellular medium. Whereas with surface-displayed PAP-Pb strain, AMP or ADP encounters with sfGFP-PAP-Pb directly on the cell surface and can be converted to ATP in the extracellular medium. Therefore, the conversion of AMP or ADP into ATP by displayed sfGFP-PAP-Pb is more efficient, resulting in a higher ATP conversion rate.

### 3.3. Effects of pH and the Concentration of Mg^2+^

**Figure 5:**
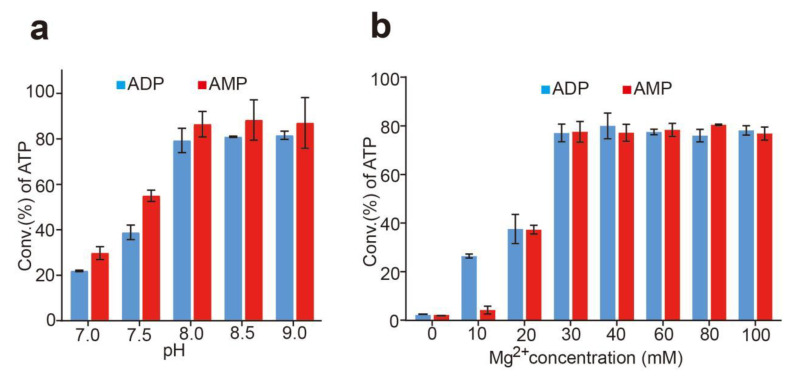
Effect of pH and the concentration of Mg^2+^ on ATP synthesis. (**a**): Effect of pH on ATP production from ADP or AMP. Productivity was assayed at 37 °C in 50 mM Tris-HCl buffer (pH from 7.0–9.0) with 20 mM poly(P), 4 mM ADP or AMP and 30 mM Mg^2+^. (**b**): Effect of the concentration of Mg^2+^ on ATP production from ADP or AMP. Productivity was assayed in 20 mM poly(P), 4 mM ADP or AMP and 50 mM Tris−HCl buffer (pH 8.5) at 37 °C with the concentration of Mg^2+^ from 0–100 mM. Results are means ± SD of three parallel replicates.

A single variable was used to determine the effects of pH and the concentration of Mg^2+^ for ATP production. As shown in Figure 5, the optimal pH was 8.5 for the synthesis reaction of ATP respectively whether using ADP or AMP as the substrate (Figure 5a). The optimal concentration of Mg^2+^ was 30 mM (Figure 5b) using ADP or AMP as the substrate, that was different to the data reported [1,12,13,17,34]. The results showed a relatively broad pH and concentration ranges of Mg^2+^ for the reaction. The conversion rate of ATP seems to be comparable when the pH ranged from 7.0 to 9.0 and the Mg^2+^ concentration ranged from 30 mM to 100 mM.

Because only one enzyme is associated with the whole reaction, the temperature, pH, and the Mg^2+^ concentration of the reaction are well controlled; moreover, a relatively broad temperature, pH, and the Mg^2+^ concentration ranges of the reaction. This ATP regeneration system is robust and convenient, so it is suitable for industrial large-scale application.

### 3.4. Effects of the Concentration of Polyphosphate

**Figure 6:**
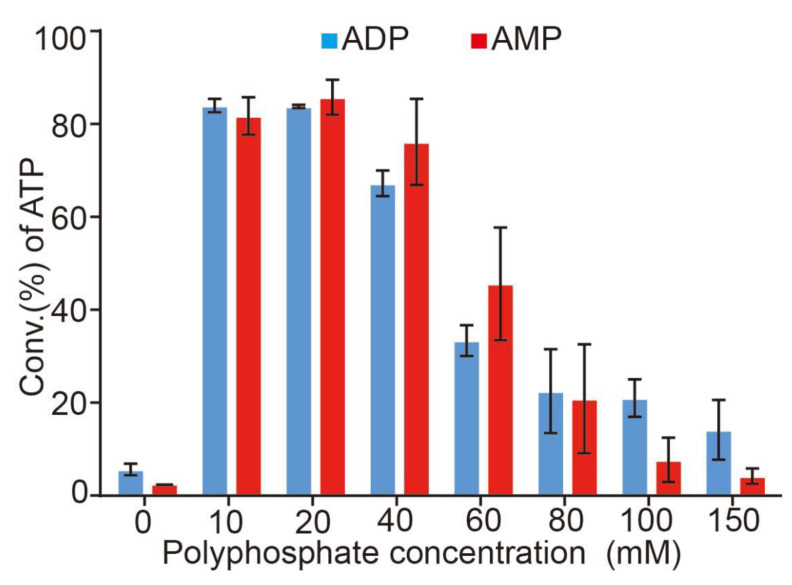
Effect of polyphosphate concentration on ATP synthesis. Productivity was assayed in 4 mM ADP or AMP, 30 mM Mg^2+^, and 50 mM Tris−HCl buffer (pH 8.5) at 37 °C with the concentration of polyphosphate from 0–150 mM. Results from three independent experiments were quantified. Error bars represent SD of three independent experiments.

In this ATP regeneration system, the adenosine scaffold could be recycled in the reaction, without a large amount of AMP or ADP [36]. While, polyphosphate as a phosphate donor, is a consumable for ATP regeneration [36]. Therefore, a large amount of polyphosphate (poly(P)) is required to improve the conversion rate of ATP regeneration. We carried out the reaction with various concentrations of poly(P) at optimal temperature, pH, and concentration of Mg^2+^. As shown in Figure 6, the optimal concentration of poly(P) was 10–20 mM for ATP regeneration. However, when the concentration of poly(P) reached to 40 mM or even more, the conversion rate of ATP regeneration dropped rapidly. The main reason may be that the high concentration of poly(P) was the inhibitor of the ATP regeneration reaction [36].

### 3.5. Effects of the Cell State on the Production of ATP

**Figure 7:**
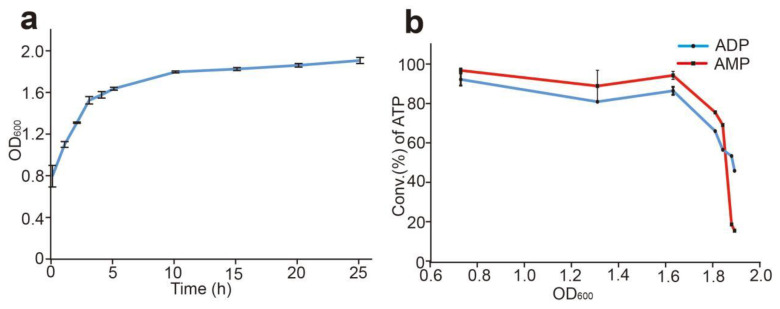
Effect of the cell state on the synthesis of ATP. (**a**): The growth curve of the sfGFP-PAP-Pb cells. (**b**): Analysis of ATP conversation using different states of the sfGFP-PAP-Pb cells. The reaction mixture contains 20 mM poly(P), 4 mM ADP or AMP, 30 mM Mg^2+^, and 50 mM Tris-HCl (pH 8.5), moderate cells were added. The total volume was bought up to 1 mL with double-distilled water. The reaction solution was incubated at 37 °C for 20 min. A small amount of reaction solution was terminated in 80 °C water bath for 10 min and diluted five-fold for HPLC analysis. Results are means ± SD of three parallel replicates.

In this reaction system, ATP was catalyzed by the intact cells of sfGFP-PAP-Pb, as described above, so the cell state should affect the synthesis reaction. In order to improve the cell performance, we performed the growth curve of cells after OD_600_ to 0.6 (Figure 7a). The same amount of cells with different OD_600_ were used in the reaction system using AMP or ADP as substrates, respectively. As shown in Figure 7b, the conversion rate of ATP maintained a higher level when the OD_600_ reached to 0.7–1.6, and the conversion rate of ATP reached to 97.5% and 80.1% respectively when AMP or ADP was used as substrates. For OD_600_ over 1.6, the conversion rate decreased rapidly.

### 3.6. Effects of Temperature and Stability of Whole-Cell Catalysts

**Figure 8:**
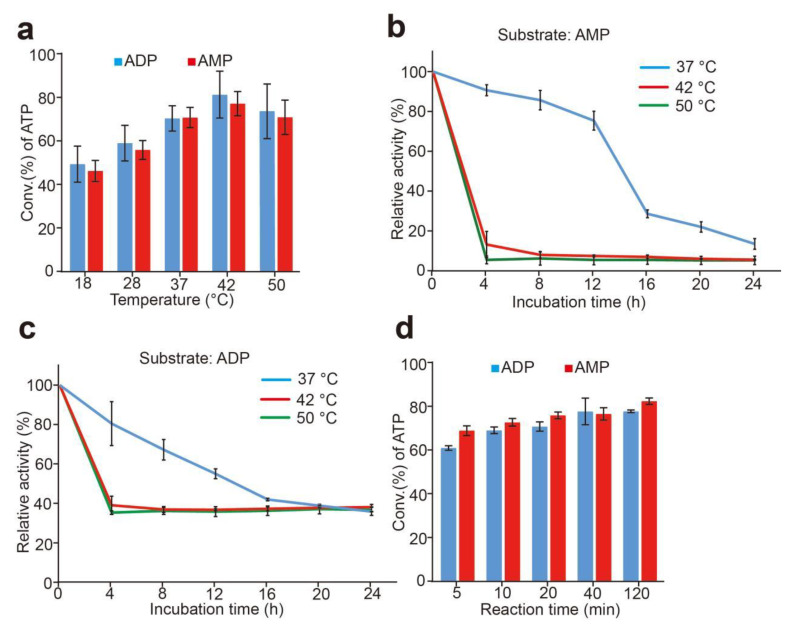
Effect of temperature on ATP synthesis and thermostability of sfGFP-PAP-Pb. (**a**): Effect of temperature on ATP production from ADP or AMP. Productivity was assayed at 18–50 °C. (**b**,**c**): Temperature dependency of the sfGFP-PAP-Pb intact cells using AMP (**b**) or ADP (**c**). The enzyme solution was incubated at 37 °C, 42 °C, and 50 °C for different times, and residual ATP-synthesizing activity was measured to determine thermostability. (**d**): Effect of reaction time on ATP production. Productivity was assayed at 37 °C for 5–120 min. The reaction mixture contains 20 mM poly(P), 4 mM ADP or AMP, 30 mM Mg^2+^ and 50 mM Tris-HCl (pH 8.5), moderate cells were added. Results are means ± SD of three parallel replicates.

The temperature result of the sfGFP-PAP-Pb for ATP production is shown in Figure 8a. Though the optimal temperature was 42 °C for the synthesis reaction of ATP when using ADP or AMP as substrates. The results showed a relatively broad temperature range for the reaction. The difference of the conversion rate of ATP is comparable when the temperature ranged from 37 °C to 50 °C.

The instability of anchoring motif on the cell surface is a critical problem of surface display in *E. coli*. Thus, we tested the stability of cell-surface-displayed sfGFP-PAP-Pb. Cells were incubated at 37 °C, 42 °C, and 50 °C for different times using ADP or AMP as the substrates respectively, and the residual activity of sfGFP-PAP-Pb was detected. As shown in Figure 8b,c, the sfGFP-PAP-Pb remained significantly stable at 37 °C. The residual activity of 78% and 60% was detected after incubation for 12 h at 37 °C when using AMP and ADP respectively. However, when at 42 °C and 50 °C, the enzyme quickly lost most of their activity. Although a little higher conversion rate of ATP at 42 °C was observed than that at 37 °C, the stability of sfGFP-PAP-Pb at 37 °C was much higher than that of 42 °C. Considering the reuse of intact cells, we chose the reaction condition at 37 °C in subsequent experiments.

Next, we examined the reaction time for the generation of ATP. The result is shown in Figure 8d, the conversion rate of ATP rose rapidly to 60% after reaction for 5 min, and reached to the plateau period when the reaction time reached 20 min.

### 3.7. Reuse of Whole-Cell Catalyst

**Figure 9:**
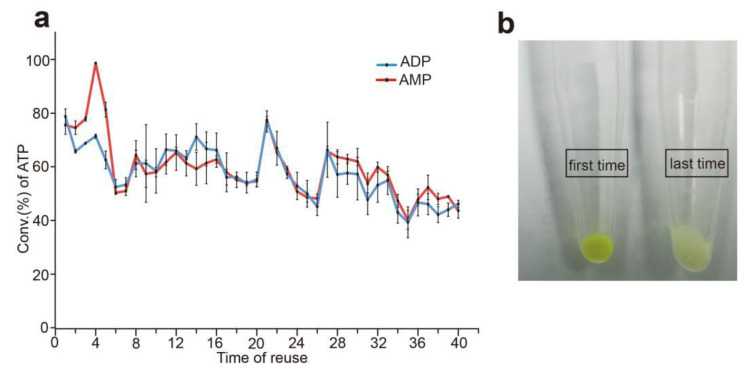
Reuse of intact cells for ATP production. (**a**): Reuse of the sfGFP-PAP-Pb intact cells. The reaction mixture contains 20 mM poly(P), 4 mM ADP or AMP, 30 mM Mg^2+^, and 50 mM Tris-HCl buffer (pH 8.5) and brought up to 1000 μL with double-distilled water, and moderate cells were added. After reaction at 37 °C for 20 min, the reaction mixture was centrifuged at 15,000 g for 5 min, and the pellet was used for the next reaction. (**b**): The green fluorescence of the sfGFP-PAP-Pb intact cells. Moderate cells were detected before and after reusing 40 times for the whole-cell catalysts.

Unlike free enzyme, intact cells as biocatalysts in the reaction mixture can be recycled as they are easily separated from the solution [17]. The fusion enzyme was displayed on the cell surface and contacted the substrate directly, and the cells could release the fresh enzyme continually. Using whole cells of *E. coli* that displayed sfGFP-PAP-Pb on the surface, we tested the possibility of reuse, as shown in Figure 9a. After a 20-min reaction, the intact cells were recovered and used as biocatalysts for the next synthesis of ATP. The conversion rate of ATP was 79.42% in the first reaction when using AMP as substrate, and after the cells were reused up to 40 times, the yield of ATP remained above 43%. When using ADP as substrate, the conversion rate of ATP was 80.51% in the first reaction, and after the cells were reused for 40 times, the residual activity was 46% of the first time.

The fusion protein with sfGFP tag has its own green fluorescence [27], which can be directly visible without any equipment, that is a very important advantage compared to other tags. Moreover, the fusion enzyme sfGFP-PAP-Pb can be displayed on the surface of the *E.* *coli* cells, so the intact cells of sfGFP-PAP-Pb are also visible with green fluorescence. As shown in Figure 9b, the green fluorescence of the intact cells became weaker after every reaction. When the fluorescence was very weak or invisible, it indicated that the intact cells were no longer available. So, we can judge from the intensity of fluorescence whether the recycle reaction should be ended without the detection of enzyme activity. That is a very convenient and economical strategy, especially for large-scale industrial applications.

## 4. Conclusions

An efficient, visible, simple, and economical method to prepare ATP from AMP or ADP with whole-cell PAP-Pb catalysts was developed by using the sfGFP to display the enzyme to the cell surface. This method is attractive because the process can be carried out without the subsequent enzyme extraction and purification steps. Moreover, only one enzyme in the reaction system and the sfGFP-PAP-Pb is visible, the visible fusion sfGFP-PAP-Pb fixed on the surface of the bacteria is very stable under the reaction condition. So, the reaction condition is easy to control, especially for large-scale industrial applications. The catalytic activity of sfGFP-PAP-Pb intact cells was 2.12 and 1.47 times higher than that of PAP-Pb intact cells, when the substrate was AMP and ADP, respectively and the conversion rate of ATP from AMP and ADP reached to 97.5% and 80.1% respectively when catalyzed by the surface-displayed enzyme at 37 °C only for 20 min. Further, the whole cell could be reused for more than 40 rounds keeping the conversion rate of ATP above 40%. Under this condition, 49.01 mg/mL (96.66 mM) ATP was accumulated for multi-rounds reaction. This resting cell reaction system could be used for ATP regeneration or coupled with other ATP-dependent enzymes for the synthesis of high-value products.

## Figures and Tables

**Table 1:** Primers used in this study.

Gene	Primer	Sequence(5′-3′)
sfGFP-PAP	SP1	GGCGGCGGCGGCAGCCATATGTTTGAAAGCGCAGAAATTG
	SP2	GCTGCCGCCGCCGCCTTTATACAGTTCATCCATGCCCAGAT
PAP	P1	CATATGTTTGAAAGCGCAGAAATTGG
	P2	GCTTTCAAACATATGATGATGATGATGATGGTGCATATGTATATCTC
sfGFP	S1	TGAGATCCGGCTGCTAACAA
	S2	AGCAGCCGGATCTCATTTATACAGTTCATCCATGCCCAG

**Table 2:** Plasmids and strains used in this study.

Strain/Plasmid	Description	Reference
pET-23a	Vector for expression proteins, T7 promoter, Amp^r^	This study
pPAP-Pb	pET-23a encoding *pap-pb*, Amp^r^	This study
psfGFP	pET-23a encoding *sfgfp*, Amp^r^	This study
psfGFP-PAP-Pb	pET-23a encoding *sfgfp*- *pap-pb*, Amp^r^	This study
Strain		
E-PAP-Pb	*E. coli* BL21(DE3) (pPAP-Pb)	This study
E-sfGFP	*E. coli* BL21(DE3) (psfGFP)	This study
E-sfGFP-PAP-Pb	*E. coli* BL21(DE3) (psfGFP-PAP-Pb)	This study

## Data Availability

All relevant data of this study are presented. Additional data will be provided upon request.
